# Characterization of *Vibrio* Populations from Cultured European Seabass and the Surrounding Marine Environment with Emphasis on *V*. *anguillarum*

**DOI:** 10.3390/microorganisms10112159

**Published:** 2022-10-31

**Authors:** Damir Kapetanović, Irena Vardić Smrzlić, Ana Gavrilović, Jurica Jug-Dujaković, Lorena Perić, Snježana Kazazić, Tea Mišić Radić, Anamarija Kolda, Milan Čanković, Jakov Žunić, Eddy Listeš, Darija Vukić Lušić, Atle Lillehaug, Semir Lončarević, Kristina Pikelj, Brigita Hengl, Dražen Knežević, Mansour El-Matbouli

**Affiliations:** 1 Ruđer Bošković Institute, 10000 Zagreb, Croatia; 2 Faculty of Agriculture, University of Zagreb, 10000 Zagreb, Croatia; 3 Sustainable Aquaculture Systems, Inc., Frenchtown, NJ 08825, USA; 4 Vodovod d.o.o., 23000 Zadar, Croatia; 5 Croatian Veterinary Institute, Regional Veterinary Institute Split, 21000 Split, Croatia; 6 Faculty of Medicine, University of Rijeka, 51000 Rijeka, Croatia; 7 Norwegian Veterinary Institute, 1433 Ås, Norway; 8 Faculty of Science, University of Zagreb, 10000 Zagreb, Croatia; 9 Croatian Agency for Agriculture and Food, 31000 Osijek, Croatia; 10 Department for Farm Animals and Veterinary Public Health, University of Veterinary Medicine Vienna, 1230 Wien, Austria; 11 School of Biotechnology, Badr University in Cairo, Cairo 11829, Egypt

**Keywords:** *Vibrio*, vibriosis, water quality, seabass, fish farm, Mali Ston Bay

## Abstract

*Vibrio* species are widely distributed and can be potentially pathogenic to aquatic organisms. In this study, we isolated *Vibrio* spp. from environmental samples (seawater, sediment, and fish swabs) collected over a three-year period from a fish farm in Mali Ston Bay in the Adriatic Sea, Croatia, and assess their distribution. A total of 48 seawater samples and 12 sediment samples, as well as gill and skin swabs from 110 farmed European seabass, were analysed for the presence of *Vibrio*. *Vibrio* strains were identified to the species level by MALDI TOF MS. The analysis revealed that *V*. *alginolyticus* was the predominant species in European seabass, followed by *V*. *anguillarum*. *V*. *alginolyticus* was isolated from the sediments, along with *V*. *gigantis* and *V*. *pomeroyi*, while *V*. *chagasii*, *V*. *cyclitrophicus*, *V*. *fortis*, *V*. *gigantis*, *V*. *harveyi*, *V*. *pelagius*, and *V*. *pomeroyi* were isolated from seawater. *V*. *anguillarum* was isolated only twice during two different spring seasons, once from a diseased sea bass and the second time from a healthy sea bass. We analysed these two isolates and found that they differ both genetically and in terms of resistance to antibiotics. Our results confirm the seasonality of vibriosis incidence and the presence of the pathogenic *V*. *anguillarum*, which increases the risk of vibriosis.

## 1. Introduction

Mediterranean countries contribute 97% of the world aquaculture production of gilthead seabream (*Sparus aurata*) and European seabass (*Dicentrarchus labrax*) [1]. In Croatia, a Mediterranean country, the European seabass is one of the most farmed fish species [2]. The occurrence of fish diseases of bacterial origin is one of the most important factors for economic losses in European seabass aquaculture [3]. One of the most significant bacterial genera considered to cause severe disease in aquacultured European seabass is the genus *Vibrio*.

The ubiquitous Vibrio are among the most abundant bacteria in marine aquatic environments worldwide [4,5] and exhibit seasonal population fluctuations [6]. The occurrence of *Vibrio* in the marine environment depends not only on parameters such as temperature, salinity, and pH [5,7] but also on organic matter enrichment, where their role is in the decomposition of organic matter [8].

Some *Vibrio* species are opportunistic pathogens that cause the most common bacterial diseases in humans and marine animals [9]. Aquacultured fish share their environment with microbiota and are therefore naturally colonised with different microbiota, which depends on the environment, water temperature, and other factors. The finding of a significant correlation between rising seawater temperature and an increase in *Vibrio* infections suggests that global warming may be a factor in the occurrence of diseases caused by *Vibrio* [5]. Some of the *Vibrio* species have been associated with health problems in aquaculture: *V*. *parahaemolyticus*, *V*. *alginolyticus*, *V*. *harveyi*, *V*. *owensii*, and *V*. *campbellii* [9]. *V*. *anguillarum* is a bacterial pathogen that causes vibriosis in fish, crustaceans, and molluscs [10,11]. Vibriosis is considered the second most important fish disease in Mediterranean aquaculture [3] and occurs as a severe infectious disease that manifests as a septic infection of the host [4,10,11]. *V*. *anguillarum* is able to adhere to fish surface cells, absorb fish mucus, and aggressively invade host epithelial and vascular tissue [10]. The resistance of *V*. *anguillarum* to antibiotics varies between strains [10] and has previously been noted with ampicillin, ciprofloxacin, erythromycin, gentamicin, nalidixic acid, tetracycline, and sulphonamides [10,12].

*Vibrio* pathogens causing diseases in aquaculture are distributed worldwide [11,13,14], and the occurrence of diseases caused by bacterial pathogens in farmed European seabass from Croatian aquaculture in the Adriatic Sea, including those caused by *V*. *anguillarum*, has already been documented in several publications [3,15,16,17,18,19].

In recent years, the ecology of pathogenic bacteria that directly or indirectly affect public health has been of great concern, especially in the context of climate change and rising ocean temperatures [5]. However, there is less information on the ecology of *V*. *anguillarum* associated with farmed European seabass and the aquaculture environment [20]. The term ecology, including the ecology of microorganisms, was coined by Ernst Haeckel (1866) and defined as the comprehensive science of the relationships of microorganisms with their environment [20,21]. The objective of this study was to determine the ecology of *V*. *anguillarum* isolates and their interactions with the other microbiota in the marine environment and with their host, European seabass, and the occurrence of *V*. *anguillarum* before and after the onset of vibriosis, together with their antibiotic resistance.

### 1.1. Sampling

**Figure 1:**
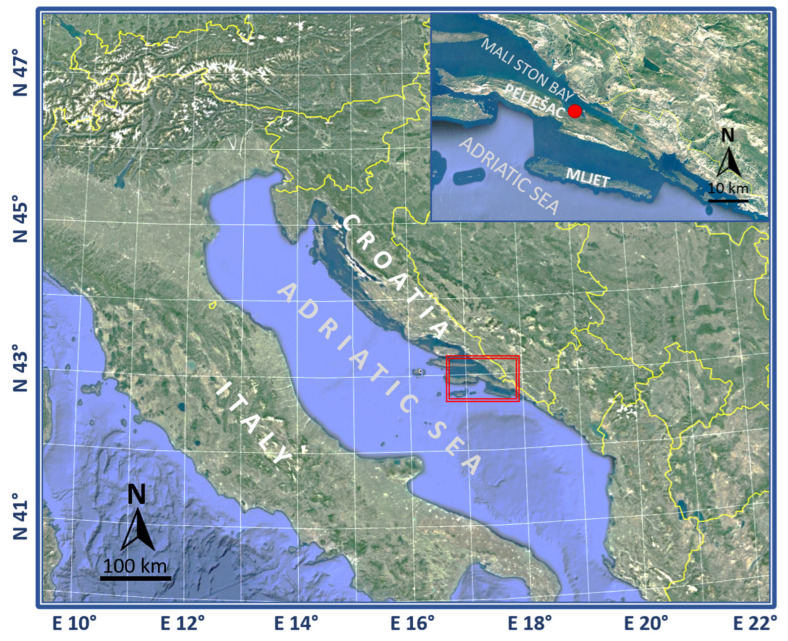
Geographic location of the investigated marine fish farm (red dot) in Mali Ston Bay in the Adriatic Sea, Croatia.

Samples of fish, seawater, and sediments were collected as part of the AQUAHEALTH project from spring 2016 to autumn 2018 (11 sampling events in the four seasons: spring, summer, fall, and winter) at the fish farm in Mali Ston Bay (42°55.0719′ N 17°29.5401′ E) in the Adriatic Sea, Croatia (Figure 1), while additional samples were collected in spring 2019 at the same farm as part of the EXAGQUA project.

The floating fish farm at a depth of approximately 19 m consists of 10-m-deep cages in which European seabass (*Dicentrarchus labrax*) and sea bream (*Sparus aurata*) are farmed. Seawater and sediment samples were collected below the cages containing European seabass, from which the fish was sampled, as well.

Seawater samples were collected at four different depths (0.5 m below the surface, 5 m deep, 10 m deep, and 0.5 m above the bottom) using a Niskin water sampler in sterilized 0.5-L plastic bottles. Sediment samples (10 g of the top sediment layer) were collected using an Ekman grab.

A total of 110 individual fish, harvested for commercial purposes, were sampled in this study. Swabs from the gills and the skin area below the dorsal fin for microbial analysis were taken using sterile rods with a 1-cm cotton tip (Deltalab, Barcelona, Spain), prior to clinical examination and health status assessment. Swab samples were diluted in 10 mL sterile phosphate-buffered saline (Sigma, St. Louis, MO, USA) in tubes and then stirred and shaken. The evaluation of the health status of the farmed fish included clinical examination and necropsy. Further, all samples were serially diluted in 10 mL of sterile PBS (Sigma, St. Louis, MO, USA), and bacterial counts were determined by the inoculation of undiluted and serially diluted samples on appropriate media.

### 1.2. Physicochemical Analysis of Seawater

Measurements of the seawater physicochemical parameters were made in situ using various portable digital probes. The SevenGo pro/Ion multiparameter probe (Mettler Toledo) was used for pH measurements. Dissolved oxygen concentration (mg/L), water oxygen saturation (%), and temperature (°C) were measured with a SevenGo pro/SG9 OptiOx probe (Mettler Toledo), while conductivity and total dissolved solids (TDS) were measured with a SevenGo pro/conductivity probe (Mettler Toledo). In the laboratory, particulate matter—seston quantity parameters’ total particulate matter (TPM), particulate organic matter (POM), and particulate inorganic matter (PIM)—were analysed using the method described by Paterson et al. [22]. Samples for the determination of the total phosphorus and nitrogen were digested using the Hach DRB200 reactor (USA). The total phosphorus and nitrogen were determined with the Hach DR6000 spectrophotometer (USA) using the Hach kit (PhosVer 3 Acigd Persulfate Digestion procedure) (USA).

### 1.3. Microbiological Analysis of Seawater and Sediment

Faecal indicators in the seawater and sediment samples were counted by a defined substrate technology using Colilert-18 (IDEXX, Westbrook, ME, USA) for the total coliform bacteria and *E*. *coli* and Enterolert-E (IDEXX) for enterococci. The total coliforms, *E*. *coli*, and enterococci were enumerated using Quantitray2000 (IDEXX), which provides the most probable numbers (MPN/100 mL).

For the enumeration of marine heterotrophic bacteria—HPC, serially diluted seawater samples were spread on Difco™ Marine Agar 2216 BD (BD, Sparks, MD, USA), and the plates were incubated at 35 °C and 22 °C for 24 h and 3–5 days, respectively.

### 1.4. Vibrio Isolation

Isolation and enumeration of *Vibrio* strains from fish gills and skin swabs, sediment, and seawater samples were performed using the plate method on a selective Thiosulfate-Citrate-Bile-Sucrose (TCBS) agar (BD). TCBS agar plates were incubated at 35 °C for 24 h and 22 °C for 3–5 days. Suspected *Vibrio* isolates that appeared as green and yellow colonies were counted, and bacterial colonies representing different morphologies per plate were selected and inoculated onto Difco™ Tryptic Soy Agar (TSA) (BD) with the addition of 1% NaCl (Kemika, Zagreb, Croatia) plates for purification and further identification by MALDI TOF MS. Results are expressed as the mean number of colony-forming units (CFU) in 1 mL of sediment and seawater or per 1 cm^2^ of gills and skin from two technical replicates by sample type.

### 1.5. Vibrio Identification Using MALDI-TOF MS

Purified presumptive *Vibrio* isolates were identified using matrix-assisted laser desorption/ionization time-of-flight mass spectrometry (MALDI-TOF MS). Sample preparation for a MALDI-TOF MS analysis followed the Bruker protocol for the extended direct transfer method. Briefly, single colonies were transferred from agar plates to a sample position on a 96-spot polished steel target plate (Bruker Daltonics, Hamburg, Germany) using a sterile wooden toothpick. Each sample was overlaid with 1 µL of 70% formic acid (Fisher Scientific, Spain) and air-dried at room temperature. Then. the sample was overlaid with 1 μL of 10 mg/mL α-cyano-4-hydroxycinnamic acid in 50% acetonitrile and 2.5% trifluoroacetic acid (Bruker Daltonics, Germany) and dried again.

Measurements were performed using a Microflex LT mass spectrometer (Bruker Daltonics, Germany), and spectra were recorded in positive linear mode within a mass range of 2–20 kDa. External calibrations were performed using the Bacterial Test Standard (Bruker Daltonics, Germany).

The recorded mass spectra were processed under the standard settings using the MALDI Biotyper Compass Explorer 4.1 software package (Bruker Daltonics, Germany). Bacteria were identified by comparing their protein mass spectral pattern with the Bruker database version 11, resulting in a logarithmic score from 0 to 3. According to the manufacturer’s recommendations, scores ≥ 2 are considered reliable species identification, from 1.7 to 1.999 are considered reliable genus identification, and scores < 1.7 are considered unreliable.

### 1.6. Confirmation of Vibrio Species Identification

For molecular identification, partial *gyrB* gene sequences from two *V*. *anguillarum* isolates were amplified, read, and compared with each other and with data from GenBank. Total DNA extracted by GenElute Mammalian Genomic kit (Sigma) from *V. anguillarum* isolates was used as the template in a further amplification assay. DNA quantity and purity were measured by a BioSpec-Nano instrument (Schimadzu, Kyoto, Japan). PCR reactions were done using the EmeraldAmpMaxHS PCR Master Kit (Promega), with the primers and cycling conditions published previously [23]. Sequencing of PCR products was performed commercially by Macrogen Europe B.V., and the sequences were deposited in GenBank under accession numbers: MK907526 and OP490254. They were analysed using the GenBank BLAST program and aligned with previously characterised gyrB sequences of the closest *Vibrio* species using ClustalX (Thompson et al. 1997). The gyrB sequences of the *V. anguillarum* strains of different geographic origin, hosts, and virulence (nonvirulent NV, low virulent LV, middle virulent MV, and high virulent HV) previously described by Castillo et al. [24], were also included in the analysis. A phylogenetic tree was constructed using MEGA version 11 software [25]. The evolutionary history was inferred by using the Maximum Likelihood method and GTR model. The robustness of the topology was checked by 1000 bootstrap replications. Accession numbers and strain names are indicated in the tree. DNAsp v.5 [26]. and MEGA 11 were used to analyse the mutation and polymorphic sites, as well as genetic diversity between Croatian isolates.

### 1.7. Atomic Force Microscopy (AFM) Imaging

For nanostructural characterisation of the *V*. *anguillarum* cell surface, topography images were collected using a Multimode scanning probe microscope with a NanoScope IIIa controller (Bruker, Billerica, MA, USA) with a vertical engagement (JV) 125-µm scanner. For the preparation of the AFM sample, 5 µL of the cell culture (0.5 McFarland) were pipetted directly onto freshly cleaved mica. Mica sheets were then placed in enclosed Petri dishes for 30–45 min to allow cells to settle and attach to the surface. Samples were then rinsed three times with ultrapure water and placed in an enclosed Petri dish, allowing the excess water to evaporate. Imaging by AFM was conducted after one hour in contact mode using silicon–nitride tips (NP-S, Bruker, nominal frequency 12–24 kHz, nominal spring constant of 0.06 N m^−1^). Measurements were done in the air at room temperature. Processing and analysis of images were carried out using NanoScope^TM^ software version 2.0 (Bruker, NanoScope Analysis, Billerica, MA, USA).

### 1.8. Antimicrobial Susceptibility

The Kirby–Bauer disk diffusion method on BBL™ Mueller–Hinton II agar (BD) was used to determine the antimicrobial susceptibility of the isolated bacterial strains. The following antimicrobial disks were used for the assay: ampicillin (10 μg), streptomycin (10 μg), gentamicin (10 μg), chloramphenicol (30 μg), ciprofloxacin (5 μg), erythromycin (15 μg), imipenem (10 μg), oxytetracycline (30 μg), sulfamethoxazole/trimethoprim (23.75/1.25 μg), and vancomycin (30 μg) manufactured by BBL™ Sensi-Disk™ and enrofloxacin (5 μg), florfenicol (30 μg), and flumequine (30 μg) manufactured by Thermo Scientific™ Oxoid. The inoculum was prepared in 5 mL of sterile 0.85% suspension medium (BioMérieux). The turbidity of each inoculum was adjusted to 0.5 according to the McFarland scale of values using the Vitek Systems ATB 1550 (BioMérieux). After 24 h of incubation at 22 °C, the diameter of the zone of inhibition was measured with a ruler, and the values were interpreted as susceptible, intermediate, or resistant, according to the manufacturer’s instructions.

The multiple antibiotic resistance index (MAR) was calculated for each tested isolate. The MAR index was calculated as a sum of the number of antibiotics to which the isolate was resistant, divided by the number of antibiotics used in this study, i.e., 13 [27].

### 1.9. Data Processing and Statistical Analyses

For each microbial indicator, an average value was calculated using data collected from the fish swabs, sediments, and water samples at four depths to provide representative information about the overall environment during four sampling periods (spring, summer, autumn, and winter). Statistical analyses (descriptive statistics, Mann–Whitney and Kruskal–Wallis tests) were performed using the statistical package SigmaPlot version 14.0 (Systat Software Inc., San Jose, CA, USA). The obtained differences were considered significant at *p* < 0.05. To visualise the *Vibrio*, present in the samples, Venn diagrams were generated using a freely available web tool (http://bioinformatics.psb.ugent.be/webtools/Venn/, accessed on 9 September 2022). A principal component analysis (PCA) was carried out by using the ‘Vegan’ package (v 2.5-7) in RStudio, version 2022.01.07 + 554 [28].

## 2. Results

### 2.1. European Seabass Health Examination

Throughout the duration of the project, the health status of the sampled European seabass was assessed at each sampling. During sampling in the spring of 2017, three seabass showed clinical signs of disease (difficulty swimming and varying degrees of petechial haemorrhage on the body surface and fin rot), which was accompanied by mortalities in the cages. On this occasion, vibriosis was suspected, and a subsequent examination of the internal organ and tissue samples from the diseased European seabass resulted in isolation of the causative agent of vibriosis, *V*. *anguillarum*. No signs of the disease were observed both before and after the outbreak of vibriosis.

### 2.2. Physicochemical Analysis of Seawater

The physicochemical parameters of the seawater were within the acceptable range for European seabass aquaculture (Table 1). The temperature exhibited seasonal variations throughout the study period, with the average water column temperature highest in the summer at 22.8 ± 1.5 °C and lowest in the winter at 12.3 ± 1.0 °C. Spring and summer sampling temperatures were significantly higher than autumn and winter sampling temperatures (*p* < 0.05), while there were no statistically significant differences in salinity between the sampling periods (*p* > 0.05). Throughout the study, the pH was lowest during winter sampling (7.9 ± 0.1), but a statistically significant difference was only found with respect to spring sampling (8.2 ± 0.2).

Dissolved oxygen in seawater at the fish farm in our study was, as expected, significantly higher in winter (10.6 ± 0.3 mg/L) than in spring, summer, and autumn (*p* < 0.05).

The average TDS value in summer and autumn was the same (25.2 mg/L). The average TDS values in spring and winter were different from those in summer and autumn, but the difference was not statistically significant (*p* > 0.05).

The highest average nitrogen content in seawater was recorded in the summer (1.3 ± 0.9 mg/L), and there was a statistically significant difference (*p* < 0.05) compared to the nitrogen content in the other three seasons, which had the same average nitrogen content of 0.6 mg/L. At the same time, the values of the second indicator of eutrophic processes, phosphorus in the seawater, differed throughout the sampling seasons, being the lowest in winter and highest in summer, but without statistical differences between them (*p* > 0.05).

### 2.3. Microbiological Analysis of Seawater and Sediment

The results of the microbiological analysis of seawater and sediment from the fish farm in Mali Ston Bay are presented in Table 2. Despite the obvious seasonal differences in the analysed microbiological parameters (total coliform bacteria, *E*. *coli*, enterococci, and HPC) in the sediment below the fish farm, the difference was not found to be statistically significant (*p* > 0.05). On the other hand, the differences in the analysed microbiological parameters, i.e., the number of total coliform bacteria in seawater were statistically significantly lower in winter than in the other sampling seasons (*p* < 0.05).

As presented in Table 2, there is no clear trend regarding the difference between seawater and sediment for certain microbiological parameters. The values for some parameters are more pronounced in seawater and, for others, in sediment. However, there is a statistically significant difference in the number of total coliforms in spring and enterococci in summer between seawater and sediment (*p* < 0.05).

### 2.4. Vibrio Counts in Gill and Skin Swabs, Seawaterm and Sediment

*Vibrio* counts were determined for the skin and gills of European seabass that were swabbed simultaneously with seawater and sediment sampling for the *Vibrio* analysis. Presumptive *Vibrio* counts tended to be higher in sediment samples than in seawater samples for all sampling periods (Table S1 and Table 3), although the differences reached statistical significance only in spring and summer for the presumptive *Vibrio* counts at 22 °C and in summer for the presumptive *Vibrio* counts at 35 °C (*p* < 0.05).

When comparing the obtained values of the presumptive *Vibrio* count for gills and skin and considering the differences between the obtained values at both incubation temperatures, there was neither a statistically significant difference between the sampling seasons nor between the obtained presumptive *Vibrio* counts on the gills and skin (*p* > 0.05). A high standard deviation of the detected levels of presumptive *Vibrio* indicates a marked variability in the *Vibrio* counts in gill and skin swabs from European seabass during the sampling session.

**Figure 2:**
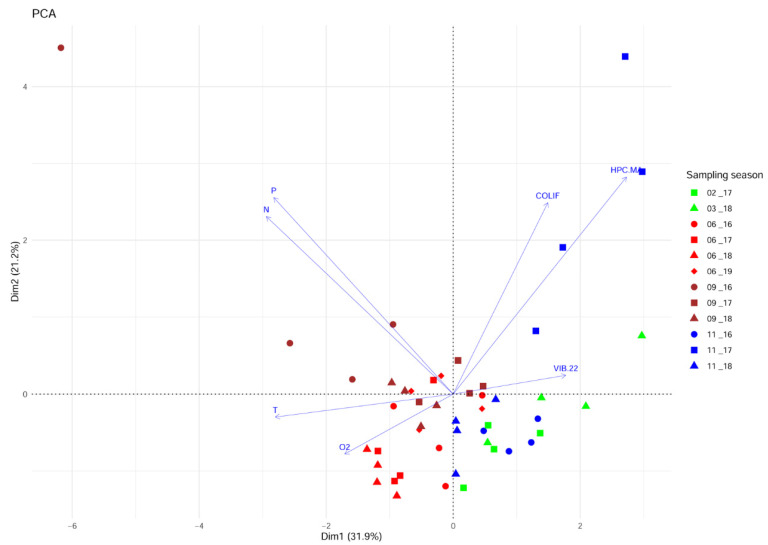
Score and variable loadings plot of the principal component analysis (PCA) based on presumptive *Vibrio* counts at an incubation temperature of 22 °C (VIB 22) and the physicochemical and microbial environmental parameters (T—temperature; O2—dissolved oxygen saturation; N—total nitrogen; P—total phosphorus; COLIF—total coliform bacteria; HPC MA—cultivable heterotrophic bacteria) in the seawater of the fish farm at Mali Ston Bay in the Adriatic Sea, Croatia.

A PCA analysis was carried out to investigate the variability of the environmental parameters’ pattern over the study period. The proportion of explained variance accounted for by the first two principal component axes (PC1 and PC2) was 53% (Figure 2). The gradient is displayed along PC1, starting from the summer and autumn samples in the left part of the biplot that correlates positively to the temperature. Among them, the cluster of samples collected in the summers of 2017 and 2018 displayed the highest values of oxygen. Negatively correlated to this group was the concentration of presumptive *Vibrio* isolated from the seawater on TCBS at 22 °C (VIB 22). The upper left part accommodates the samples associated with the total phosphorus (P) and total nitrogen (N) concentrations. These variables were negatively correlated with the autumn and winter samples distributed in the lower right part of the biplot. The samples collected in November 2017 are distributed in the upper right part of the biplot and linked to the concentration of coliforms (COLIF) and cultivable heterotrophic bacteria (HPC.MA). PCA ordination carried out for characterisation of the sampling area using data collected over the whole study period showed that P, N, COLIF, HPC.MA, and T were the most important variables for ordination.

### 2.5. Vibrio Identification

**Figure 3:**
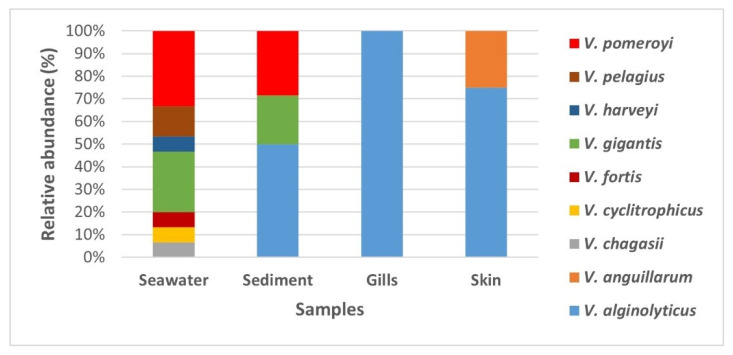
Relative abundance of the bacterial genera isolated and identified in each sample type: seawater, sediment, and swabs of European seabass gills and skin from the fish farm at Mali Ston Bay in the Adriatic Sea.

The MALDI TOF MS analysis of presumptive *Vibrio* isolates (Table S2) revealed that the isolates predominantly belonged to *Vibrio* genera (Figure 3). Among the *Vibrio* genera, nine *Vibrio* species were identified (*V*. *alginolyticus*, *V*. *anguillarum*, *V*. *chagasii*, *V*. *cyclitrophicus*, *V*. *fortis*, *V*. *gigantis*, *V*. *harveyi*, *V*. *pelagius*, and *V*. *pomeroyi*). Their abundance differed in the different sample types.

**Figure 4:**
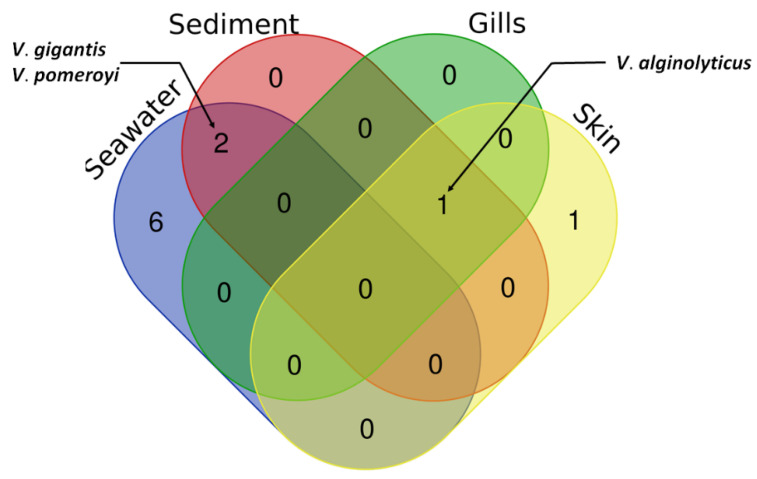
Venn diagram of *Vibrio* species identified from cultivable microbiota of seawater, sediment, and swabs of European seabass gills and skin from a fish farm at Mali Ston Bay in the Adriatic Sea.

The greatest diversity of bacterial species was found in the seawater samples, a total of eight species, two of which were common within sediment samples (*V*. *gigantis* and *V*. *pomeroyi*) (Figure 4). On the other hand, three species were detected in the sediment samples (*V*. *alginolyticus*, *V*. *gigantis*, and *V*. *pomeroyi*). Interestingly, *V*. *algynoliticus* was the only strain isolated from gills. It was also predominant on the skin, comprising >70% of isolates, whereas the rest of skin swab isolates belonged to *V*. *anguillarum* (Figure 4), the known causative agent of furunculosis in European seabass in aquaculture worldwide.

### 2.6. Characterisation of V. anguillarum Isolates

Two *V*. *anguillarum* isolates from diseased (spring 2017) and healthy (spring 2019) European seabass from a fish farm in Mali Ston Bay were characterised using molecular methods: atomic force microscopy (AFM) and the antimicrobial susceptibility test.

**Figure 5:**
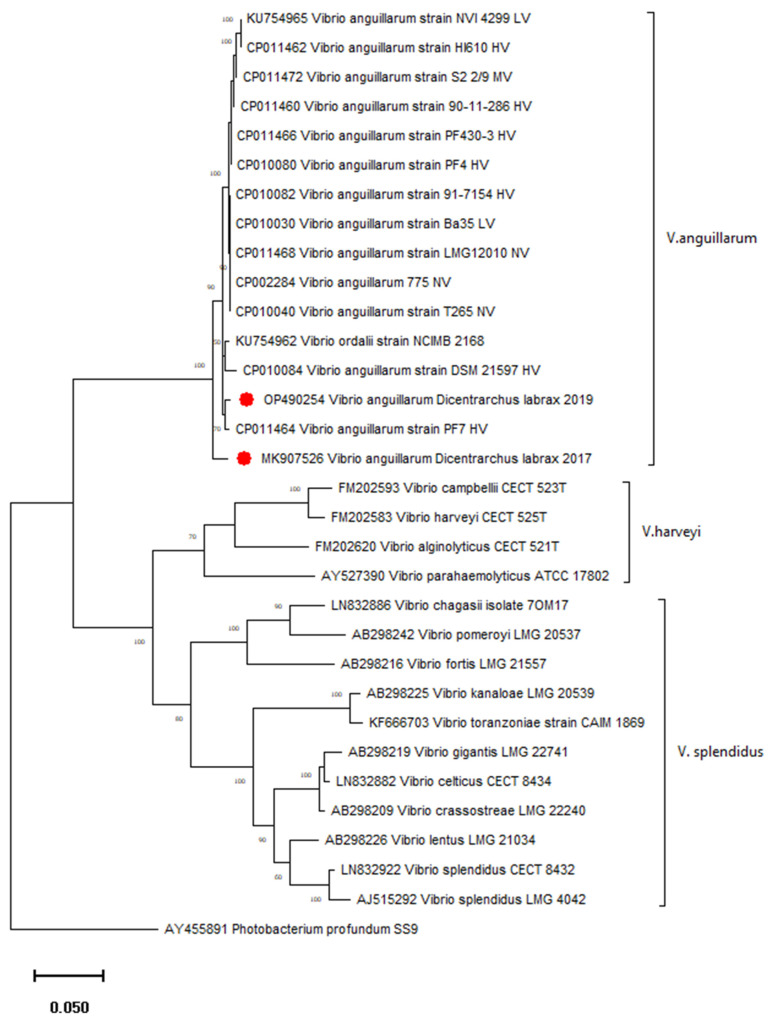
Phylogenetic tree based on partial *gyrB* gene sequences from Croatian (●) and previously published close *Vibrio* strains. The tree was inferred by using the Maximum Likelihood method and GTR model. Bootstrap values lower than 70 were removed from the tree. The horizontal bar at the base of the figure represents 0.05 substitutions per amino acid site. The virulence ranking of the *V. anguillarum* strains was published previously: HV, high virulence; LV, low virulence; MV, medium virulence [24].

The sequence analysis of the *gyrB* gene confirmed *V*. *anguillarum* species and showed 2.4% of the divergence between two Croatian strains isolated from European seabass in 2017 and 2019. Their nucleotide diversity (π) was 0.02174, and the number of mutations, as well as polymorphic sites, was 19. A phylogenetic tree confirmed separate groupings of different genetic clusters of the genus *Vibrio* (Figure 5.). The *V*. *anguillarum* strain isolated in 2019 from healthy seabass clusters with high virulent strains from vibriosis outbreaks in farmed fish (Figure 5). The *V. anguillarum* strain isolated in 2017 from diseased seabass was phylogenetically separated from other strains included in the tree (Figure 5). Accordingly, the *V*. *anguillarum* strain from 2017 showed higher divergence (2.18–2.75%) from the other representative strains from GenBank, while this divergence for the *V*. *anguillarum* strain isolated in 2019 was lower (0.75–2.4%).

**Figure 6:**
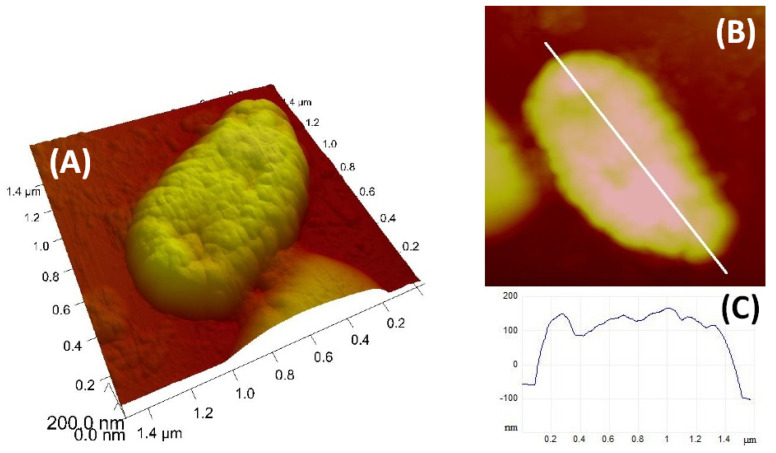
AFM image of *V*. *anguillarum* (scan size: 1.5 µm × 1.5 µm, vertical scale 200 nm). Three-dimensional height data: (**A**) top view of the height data (**B**) and with the corresponding vertical profile along the indicated line (**C**).

Nanostructural characterisation of the *V*. *anguillarum* cell was performed by AFM. Representative images of individual cells are shown in Figure 6. The *V*. *anguillarum* cells have ellipsoid shapes with a length 1.4 µm, width 750 nm, and height 250 nm, and the surface of the cell shows a granular structure.

### 2.7. Antimicrobial Resistance of V. anguillarum Isolates

The antimicrobial activity of 13 antibiotics was determined against *V*. *anguillarum* strains isolated from diseased and healthy European seabass. The tested antibiotics were classified according to their activity (Table 4).

Both isolates of *V*. *anguillarum* showed antimicrobial resistance to ampicillin, erythromycin, and vancomycin. The isolated strain obtained from diseased European seabass during the vibriosis outbreak was also resistant to imipenem and ciprofloxacin.

The pattern of antibiotic resistance was highest in the *V*. *anguillarum* strain isolated from diseased European seabass, with a multiple antibiotic resistance index (MAR) of 0.38. In contrast, the strain isolated from healthy European seabass had multiple resistances to three of the thirteen antibiotics used and, consequently, had a MAR index of 0.23.

## 3. Discussions

Environmental characteristics of the marine environment that define the carrying capacity required for aquaculture sustainability, including, among other properties, the physicochemical and microbiological properties of seawater and sediment [29]. Physicochemical and microbiological properties of seawater and sediment at the fish farm in Mali Ston Bay were consistent with previous studies in the Adriatic Sea carried out at similar floating fish farms [2,4].

Although the principal components captured roughly only half of the total variance, the obvious gradients and clusters of samples indicated the substantial influence of fluctuating environmental factors over the sampling period. The seasonality pattern was primarily detectable by the gradient from the spring/summer to autumn/winter samples visible along the first PC axis (Figure 2). Despite the lower importance for the ordination of presumptive *Vibrio* isolated at 22 °C, its inverse trend with the temperature might be related to thermal optimums for *Vibrio* species from the water column whose density was negatively correlated with the temperature [20]. The microbiological quality and nutrient enrichment were only occasionally disrupted.

It is well-known that outbreaks of fish diseases are a result of the interaction between the pathogen, the host, and the environment [29]. For this reason, we based our research on the results of a multi-year study of the presence of fish pathogens of the genus *Vibrio* in seawater, sediment, and fish swab samples from 12 samplings, seasonally from spring 2016 to autumn 2018, and additionally in spring 2019.

A vibriosis of European seabass caused by the bacterium *V*. *anguillarum* occurred at the fish farm in spring 2017. *V*. *anguillarum* is known to be isolated from environmental samples—not only from fish samples but also from seawater and sediment [11]. However, during our four pre-disease samplings, *V*. *anguillarum* was not isolated from any of the samples (seawater, sediment, fish gill, and skin swabs). After the outbreak of vibriosis, the pathogen *V*. *anguillarum* was not isolated in the next six samplings until spring 2019 when it was isolated again from a skin swab of a healthy European seabass. We analysed these two strains of *V*. *anguillarum* and the seawater and sediment samples to investigate the ecology of the *V*. *anguillarum* isolates. Other studies also emphasised that *Vibrio* spp. were relatively more abundant in sediment than in water, suggesting that the sediment may serve as a reservoir for *Vibrio* spp., especially in winter [5]. The results of this study partly indicate a situation previously recorded in the Adriatic Sea in a similar farm, where *V*. *alginolyticus* was the only *Vibrio* species isolated from the fish and seawater [4]. *V*. *alginolyticus* was not detected in seawater during the sampling period; however, it was the only *Vibrio* isolated from gill swabs, whereas *V*. *alginolyticus* and *V*. *anguillarum* were isolated from the skin of diseased and healthy European seabass. The identity of these two *V*. *anguillarum* isolates from diseased and healthy European seabass, previously identified with MALDI TOF MS, was confirmed by a partial *gyrB* sequence analysis; moreover, the molecular analysis revealed a notable genetic diversity between these two strains, despite the same geographical origin and the same host species. Although a previous study of *V. anguillarum* genomes reported clustering at some extent of different strains from the same host species and/or geographical origin [24], great diversity within 39 *V. anguillarum* virulent strains from rainbow trout in Denmark was found recently [30]. In terms of virulence, a comparison of the *V. anguillarum* strains based on the core genome diversity indicated that medium to nonvirulent strains grouped together due to their low genetic diversity, while the most virulent strains grouped into different genetic clusters [24]. The strain isolated in the present study from clinically healthy seabass clusters with high virulent strains from Norway and Chile, distantly from the group of middle to nonvirulent strains, while the strain isolated from diseased fish represents a separated branch. Although we cannot make conclusions about the degree of virulence of *V. anguillarum* isolates on the basis of our results, their rather high genetic divergence is significant. A comparative genome analysis should provide more insights into their genetic diversity, as our results are based only on the *gyrB* gene. As investigated strains are isolated in different years, there is a possibility that one or more constant sources for the spreading of bacteria are present. Various factors, particularly the origin of fish and environmental factors (including water quality and farm management), as well as surrounding wild fish populations, could influence the occurrence of *V. anguillarum*.

Since AFM readily provides images of natural bacterial cells at high magnification in three dimensions, it can be used as a tool to study the fine structures of marine bacteria [31]. According to the available literature, this is the first AFM image of *V*. *anguillarum* that provides information on the bacterial size and cell surface structure.

The problem of bacterial resistance to antibiotics is of great importance for the ecology of microorganisms and their health risk [32]. The detected genetic differences of *V*. *anguillarum* isolates were confirmed with the differences in their resistance to antibiotics. Considering the ecology and potential pathogenicity of *V*. *anguillarum*, it is important to highlight its antibiotic resistance, which differs among these isolates, as confirmed by the difference in the multiple antibiotic resistance index (MAR). The isolate from diseased European seabass had a MAR index of 0.38, while the isolate from healthy European seabass had an index of 0.23. Hickey and Lee [10] and Parin et al. [12] reported multiple antibiotic resistances of *V*. *anguillarum* to several antibiotics (ampicillin, ciprofloxacin, erythromycin, gentamicin, nalidixic acid, tetracycline, and sulphonamides). All these results highlighted the problem of antibiotic resistance among bacterial pathogens in aquaculture, as well as the difficulties in treating diseased fish and the importance of targeting therapy only with the most appropriate antimicrobials or, even better, introducing alternative treatments such as “phage therapy” and sustainable control measures to overcome the risk developing and spreading of antibiotic resistance [33].

In conclusion, this study suggests that sediments may be an important reservoir for *Vibrio* spp. The results confirm that farmed European seabass carry significant numbers of *V*. *alginolyticus*. However, *V*. *alginolyticus* was found in sediments rather than seawater. This study demonstrates the presence of the potentially pathogenic *V*. *anguillarum* as part of the cultivable microbiota of farmed European seabass. *V*. *anguillarum* is an important fish pathogen, and several methods were used in this study to observe the differences between isolates. Our study revealed genetic differences among *V*. *anguillarum* isolates, and we found differences in antimicrobial resistance among them. The reason for these differences remains to be determined but could be related to the different origins of the isolates. The results also provide evidence of multiple antibiotic resistance in strains of *V*. *anguillarum* isolated from European seabass in Croatia. This indicates the need for the continuous monitoring and application of strategies to reduce antibiotic use in aquaculture and the need for alternative phage therapy.

## Figures and Tables

**Table 1:** Physicochemical analysis of seawater on the fish farm at Mali Ston Bay in the Adriatic Sea, Croatia.

	Spring				Summer				Autumn				Winter			
	Average	SD	Min	Max	Average	SD	Min	Max	Average	SD	Min	Max	Average	SD	Min	Max
Temperature (°C)	21.3	2.3	18.3	25.2	22.8	1.5	20.9	24.9	18.0	2.1	15.0	19.9	12.3	1.0	10.1	12.9
Salinity (‰)	33.0	3.4	26.8	38.3	33.3	1.5	30.6	34.9	34.3	1.0	33.5	37.0	32.3	3.1	24.9	34.7
pH	8.2	0.2	7.9	8.5	8.0	0.2	7.8	8.3	8.0	0.2	7.6	8.3	7.9	0.1	7.7	7.9
Dissolved oxygen (mg/L)	8.9	1.2	6.6	10.6	8.7	0.6	7.3	9.3	9.1	0.5	8.6	9.9	10.6	0.3	10.1	11.1
Oxygen saturation (%)	105.4	5.1	96.3	115.1	100.8	6.3	84.1	108.4	96.2	2.7	91.8	99.7	101.1	3.2	95.6	107.0
TDS (mg/L)	24.5	1.9	20.9	26.7	25.2	1.7	20.6	26.5	25.2	3.1	15.9	28.3	24.9	2.2	19.8	26.7
N (mg/L)	0.6	0.4	0.1	1.6	1.3	0.9	0.6	3.6	0.6	0.3	0.09	0.98	0.6	0.1	0.4	0.8
P (mg/L)	0.04	0.03	0.01	0.1	0.09	0.2	0.01	0.6	0.03	0.02	0.01	0.07	0.01	0.007	0.01	0.03

SD—standard deviation; Min—minimum value; Max—maximum value; TDS—total dissolved solids; N—total Nitrogen; P—total Phosphorus.

**Table 2:** Microbiological analysis of seawater and sediment from fish farm at Mali Ston Bay in the Adriatic Sea, Croatia.

	Spring		Summer		Autumn		Winter	
	Seawater	Sediment	Seawater	Sediment	Seawater	Sediment	Seawater	Sediment
TC (MPN/100 mL)	255.2 ± 259.0	30.6 ± 31.8	306.1 ± 270.3	521.7 ± 822.2	582.9 ± 774.5	1224.3 ± 2015.4	13.1 ± 7.1	10.0 ± 0
EC (MPN/100 mL)	15.0 ± 15.2	9.9 ± 0.1	48.0 ± 58.0	22.0 ± 16.6	15.6 ± 13.7	572.1 ± 923.5	9.9 ± 0	9.9 ± 0
ENT (MPN/100 mL)	10.2 ± 1.3	10.8 ± 5.6	10.8 ± 2.9	62.5 ± 78.0	51.1 ± 67.7	27.3 ± 25.9	10.6 ± 1.8	9.9 ± 0
HPC (CFU/mL)	1290.6 ± 936.5	2478.8 ± 933.8	1742.5 ± 949.7	2696.7 ± 1391.4	3495.0 ± 3186.7	2860.0 ± 1607.3	10.6 ± 1659.9	3368.5 ± 832.3

Values are the average ± standard deviation; TC—total coliform bacteria; EC—*E. coli*; ENT—enterococci; HPC—heterotrophic bacteria.

**Table 3:** Presumptive *Vibrio* counts during the four sampling seasons in seawater, sediment, and gills and skin swabs of European seabass in the fish farm at Mali Ston Bay in the Adriatic Sea, Croatia.

	Spring	Summer	Autumn	Winter
*Vibrio* 22 °C (CFU/mL)				
Seawater	25.9 ± 22.6	93.3 ± 113.5	109.6 ± 132.8	280.3 ± 341.5
Sediment	225.8 ± 113.6	360.0 ± 295.5	365.7 ± 250.6	696.0 ± 825.9
Gills	16.8 ± 62.4	1.2 ± 3.2	90.5 ± 271.9	5.4 ± 15.4
Skin	7.1 ± 25.6	0 ± 0	4.8 ± 21.8	2.6 ± 9.8
*Vibrio* 35 °C (CFU/mL)				
Seawater	7.1 ± 10.8	4.4 ± 7.0	14.3 ± 40.6	0 ± 0
Sediment	14.5 ± 17.2	179.0 ± 129.7	11.3 ± 10.3	0 ± 0
Gills	4.6 ± 17.4	0 ± 0	0 ± 0	0 ± 0
Skin	3.5 ± 12.3	0 ± 0	0 ± 0	0.2 ± 0.7

**Table 4:** Antibiotic resistance profile of two *V*. *anguillarum* strains isolated from European seabass at a fish farm at Mali Ston Bay in the Adriatic Sea, Croatia.

	*V. anguillarum*	
Antibiotic	Strain Spring 2017	Strain Spring 2019
Enrofloxacin	S	I
Florfenicol	S	S
Gentamicin	S	S
Ampicillin	R	R
Erythromycin	R	R
Oxytetracycline	I	I
Sulfamethoxazole/trimethoprim	S	S
Vancomycin	R	R
Flumequine	S	S
Imipenem	R	S
Ciprofloxacin	R	I
Streptomycin	I	I
Chloramphenicol	S	S

S—Susceptible; I—Intermediate; R—Resistant.

## Data Availability

The data presented in this study are available on request from the corresponding author.

## Supplementary Materials

The following supporting information can be downloaded at: https://www.mdpi.com/article/10.3390/microorganisms10112159/s1. Table S1. Presumptive *Vibrio* counts (CFU/mL) during four sampling seasons (spring, summer, autumn, winter) in seawater, sediment, and gill and skin swabs of European seabass in the fish farm in Mali Ston Bay in the Adriatic Sea, Croatia, determined in the period 2016 to 2019. Table S2. Raw data of MALDI-TOF MS identification of *Vibrio* species using the MALDI Biotyper Compass Explorer 4.1 software package and Bruker database version 11.
