# Supplementary material for: Characterization of Vibrio Populations from Cultured European Seabass and the Surrounding Marine Environment with Emphasis on V. anguillarum
Source: Microorganisms. 2022 Oct 31;10(11):2159. doi: 10.3390/microorganisms10112159 (PMC9695460; doi:10.3390/microorganisms10112159)
Supplement: Supplementary file 1 [file microorganisms-10-02159-s001.zip › microorganisms-1948119-supplementary.pdf]

Table S1. Presumptive *Vibrio* counts (CFU/mL) during four sampling seasons (spring, summer, autumn, winter) in seawater, sediment, and gill and skin swabs of European seabass in the fish farm in Mali Ston Bay in the Adriatic Sea, Croatia, determined in the period 2016 to 2019.

		Spring	Summer	Autumn	Winter
**2016**	*Vibrio* 22 °C				
	Seawater	25.0 ± 24.9	44.3 ± 52.4	170.0 ± 130.8	na
	Sediment	220.0 ± 45.3	165.0 ± 9.9	177.0 ± 4.2	na
	Gills	0 ± 0	0 ± 0	0 ± 0	na
	Skin	0 ± 0	0 ± 0	0 ± 0	na
	Vibrio 35 °C				
	Seawater	1.0 ± 1.4	0.25 ± 0.5	43.0 ± 66.3	na
	Sediment	0 ± 0	42.0 ± 9.9	20.0 ± 0	na
	Gills	0 ± 0	0 ± 0	0 ± 0	na
	Skin	0 ± 0	0 ± 0	0 ± 0	na
**2017**	*Vibrio* 22 °C				
	Seawater	13.3 ± 11.3	170.0 ± 175.1	35.0 ± 56.9	60.5 ± 38.2
	Sediment	345.0 ± 35.4	700.0 ± 113.1	270.0 ± 11.3	1280.0 ± 113.1
	Gills	52.0 ± 119.2	3.3 ± 4.8	0 ± 0	10.8 ± 20.8
	Skin	13.7 ± 34.3	0 ± 0	0 ± 0	0 ± 0
	Vibrio 35 °C				
	Seawater	5.0 ± 10.0	5.25 ± 9.9	0 ± 0	0 ± 0
	Sediment	24.0 ± 8.5	195.0 ± 18.4	0 ± 0	0 ± 0
	Gills	43.4 ± 89.1	0 ± 0	0 ± 0	0 ± 0
	Skin	13.3 ± 22.4	0 ± 0	0 ± 0	0 ± 0
**2018**	*Vibrio* 22 °C				
	Seawater	50.0 ± 19.6	65.5 ± 41.6	123.8 ± 178.4	500.0 ± 376.6
	Sediment	264.0 ± 104.7	215.0 ± 35.4	650.0 ± 70.7	112.0 ± 4.2
	Gills	0.5 ± 0.7	0.1 ± 0.3	237.5 ± 413.8	0 ± 0
	Skin	0 ± 0	0 ± 0	12.5 ± 35.4	5.2 ± 13.7
	Vibrio 35 °C				
	Seawater	22.5 ± 6.5	7.75 ± 6.6	0 ± 0	0 ± 0
	Sediment	34.0 ± 22.6	300.0 ± 28.3	14.0 ± 1.4	0 ± 0
	Gills	0 ± 0	0 ± 0	0 ± 0	0 ± 0
	Skin	0 ± 0	0 ± 0	0 ± 0	0.3 ± 0.9
**2019**	*Vibrio* 22 °C				
	Seawater	15.3 ± 16.8	na	na	na
	Sediment	74.0 ± 9.9	na	na	na
	Gills	14.6 ± 26.1	na	na	na
	Skin	14.6 ± 37.9	na	na	na
	Vibrio 35 °C				
	Seawater	0 ± 0	na	na	na
	Sediment	0 ± 0	na	na	na
	Gills	0 ± 0	na	na	na
	Skin	0.8 ± 2.5	na	na	na

Values are average ± standard deviation; na – not analysed

Table S2. Raw data of MALDI-TOF MS identification of *Vibrio* species using the MALDI Biotyper Compass Explorer 4.1 software package and Bruker database version 11.

Sampling year	Tested isolates	Bruker database	
		Identification	Log score
2016	VP 1	*V. pomeroyi*	2.040
	VP 2	*V. pomeroyi*	1.840
	VP 3	*V. pomeroyi*	2.100
	VP 4	*V. chagasii*	1.970
	VP 5	*V. gigantis*	2.080
	VP 6	*V. ichthyoenteri*	2.070
	VP 7	*V. gigantis*	2.040
	VP 8	*V. pomeroyi*	1.940
	VP 10	*V. ichthyoenteri*	1.930
	VP 11	*V. pomeroyi*	2.140
	VP 12	*V. chagasii*	1.880
	VP 13	*V. pomeroyi*	2.090
	VP 14	*V. pomeroyi*	2.010
	VP 15	*V. ichthyoenteri*	2.060
	VP 16	*V. pomeroyi*	1.990
	VP 17	*V. ichthyoenteri*	1.880
	VP 18	*V. gigantis*	1.980
	VP 22	*V. harveyi*	2.050
	VP 23	*V. ichthyoenteri*	1.990
	VP 24	*V. scophthalmi*	1.940
	VP 25	*V. harveyi*	2.210
	VP 26	*V. harveyi*	2.300
	LJV 16 5	*V. pomeroyi*	1.910
	LJV 16 6	*V. pomeroyi*	1.950
	LJV 16 7	*V. pomeroyi*	2.040
	LJV 16 10	*V. gigantis*	2.100
	LJV 16 11	*V. superstes*	1.830
	LJV 16 15	*V. pomeroyi*	2.130
	LJV 16 19	*V. gigantis*	2.120
	LJV 16 20	*V. superstes*	1.890
	LJV 16 21	*V. gigantis*	1.920
	LJV 16 22	*V. pomeroyi*	2.040
	LJV 16 23	*V. pomeroyi*	2.040
	LJV 16 25	*V. harveyi*	1.810
	LJV 16 26	*V. harveyi*	2.240
	LJV 16 28	*V. harveyi*	2.300
	LJV 16 30	*V. harveyi*	1.910
	LJV 16 31	*V. superstes*	1.760
	VJ 16 6	*V. cyclitrophicus*	2.200
	VJ 16 7	*V. gigantis*	1.990
	VJ 16 8	*V. pomeroyi*	1.950
	VJ 16 9	*V. pomeroyi*	1.900
	VJ 16 12	*V. gigantis*	1.940
	VJ 16 13	*V. pomeroyi*	2.060
	VJ 16 14	*V. pomeroyi*	1.980
	VJ 16 16	*V. harveyi*	2.470
	VJ 16 17	*V. harveyi*	2.430
	VJ 16 18	*V. harveyi*	2.450
	VJ 16 19	*V. orientalis*	1.960
	VJ 16 20	*V. harveyi*	2.260

	VJ 16 21	*V. harveyi*	2.410
	VJ 16 22	*V. harveyi*	2.380
	VJ 16 23	*V. harveyi*	2.280
	VJ 16 24	*V. harveyi*	2.210
	VJ 16 25	*V. europaeus*	2.120
	VJ 16 26	*V. pelagius*	1.850
	VJ 16 31	*V. gigantis*	1.830
	VJ 16 32	*V. gigantis*	1.970
**2017**	P17 21	*V. harveyi*	1.920
	P17 22	*V. fortis*	1.860
	P17 24	*V. gigantis*	1.890
	P17 26	*V. pomeroyi*	1.920
	P17 29	*V. pomeroyi*	1.870
	P17 32	*V. pomeroyi*	1.930
	P17 111	*V. alginolyticus*	1.830
	P17 112	*V. alginolyticus*	2.080
	P17 113	*V. alginolyticus*	1.870
	P17 114	*V. alginolyticus*	2.010
	P17 126	*V. alginolyticus*	1.840
	P17 127	*V. alginolyticus*	2.110
	P17 128	*V. alginolyticus*	2.260
	P17 129	*V. alginolyticus*	2.100
	P17 130	*V. alginolyticus*	1.900
	P17 131	*V. alginolyticus*	2.060
	P17 132	*V. alginolyticus*	2.040
	P17 137	*V. alginolyticus*	2.000
	P17 138	*V. alginolyticus*	2.100
	P17 139	*V. alginolyticus*	2.000
	P17 140	*V. alginolyticus*	2.150
	P17 143	*V. alginolyticus*	1.890
	P17 144	*V. alginolyticus*	2.090
	P17 155	*V. alginolyticus*	2.150
	P17 9	*V. alginolyticus*	2.040
	P17 10	*V. alginolyticus*	1.970
	P17 11	*V. anguillarum*	2.370
	P17 12	*V. anguillarum*	2.330
	P17 13	*V. anguillarum*	2.140
	P17 14	*V. anguillarum*	2.380
	P17 15	*V. anguillarum*	2.290
	P17 16	*V. anguillarum*	2.280
	ZM17 35	*V. tasmaniensis*	1.980
	ZM17 36	*V. tasmaniensis*	1.870
	ZM17 38	*V. pelagius*	1.870
**2018**	P18 37	*V. alginolyticus*	2.100
	P18 48	*V. alginolyticus*	2.110
	P18 64	*V. scophthalmi*	2.120
	P18 65	*V. pomeroyi*	2.070
	P18 68	*V. harveyi*	2.310
	P18 70	*V. harveyi*	2.200
	P18 71	*V. harveyi*	2.330
	P18 73	*V. harveyi*	2.420
	LJ18 17	*V. chagasii*	1.880
	LJ18 18	*V. pomeroyi*	1.950

LJ18 20	*V. alginolyticus*	2.040
LJ18 21	*V. alginolyticus*	2.170
LJ18 22	*V. alginolyticus*	1.940
LJ18 23	*V. alginolyticus*	2.110
LJ18 24	*V. alginolyticus*	2.180
LJ18 25	*V. harveyi*	2.200
LJ18 26	*V. harveyi*	2.150
LJ18 27	*V. alginolyticus*	2.220
LJ18 29	*V. alginolyticus*	2.140
LJ18 31	*V. harveyi*	2.280
LJ18 33	*V. harveyi*	2.180
LJ18 34	*V. chagasii*	2.000
LJ18 35	*V. chagasii*	2.000
LJ18 59	*V. harveyi*	2.210
LJ18 60	*V. harveyi*	2.040
LJ18 62	*V. pelagius*	2.190
LJ18 65	*V. pelagius*	2.230
LJ18 66	*V. chagasii*	1.770
LJ18 69	*V. chagasii*	1.870
LJ18 138	*V. chagasii*	1.890
LJ18 139	*V. fortis*	2.300
LJ18 141	*V. gigantis*	2.090
LJ18 142	*V. chagasii*	2.050
LJ18 143	*V. harveyi*	2.120
LJ18 144	*V. chagasii*	1.980
LJ18 149	*V. pomeroyi*	1.980
LJ18 150	*V. chagasii*	1.950
LJ18 153	*V. gigantis*	2.040
LJ18 154	*V. gigantis*	2.030
LJ18 155	*V. chagasii*	2.060
LJ18 156	*V. chagasii*	2.080
LJ18 157	*V. chagasii*	2.090
J18 9	*V. harveyi*	2.150
J18 10	*V. harveyi*	1.900
J18 11	*V. harveyi*	2.100
J18 12	*V. harveyi*	2.160
J18 13	*V. gigantis*	2.080
J18 14	*V. pomeroyi*	1.920
J18 16	*V. pomeroyi*	2.020
J18 17	*V. pomeroyi*	1.830
J18 18	*V. pomeroyi*	1.890
J18 19	*V. gigantis*	1.860
J18 20	*V. gigantis*	1.780
J18 21	*V. pomeroyi*	2.020
J18 22	*V. gigantis*	2.030
J18 23	*V. pomeroyi*	1.970
J18 24	*V. pomeroyi*	2.020
J18 26	*V. alginolyticus*	2.080
J18 28	*V. alginolyticus*	1.950
J18 29	*V. harveyi*	2.040
J18 31	*V. alginolyticus*	1.970
J18 32	*V. alginolyticus*	2.330
Z18 41	*V. tasmaniensis*	1.950
Z18 42	*V. tasmaniensis*	1.850
Z18 45	*V. tasmaniensis*	1.980

	Z18 46	*V. tasmaniensis*	1.960
	Z18 49	*V. tasmaniensis*	1.890
	Z18 69	*V. tasmaniensis*	1.950
	Z18 71	*V. tasmaniensis*	1.900
	Z18 110	*V. tasmaniensis*	1.860
	Z18 120	*V. tasmaniensis*	1.990
**2019**	14	*V. anguillarum*	2.280
	22	*V. alginolyticus*	1.870
	24	*V. alginolyticus*	2.160
	27	*V. chagasii*	1.710
	28	*V. alginolyticus*	2.150
	29	*V. chagasii*	1.890
	30	*V. orientalis*	1.830
	31	*V. orientalis*	1.940
	33	*V. harveyi*	2.120
	35	*V. pomeroyi*	1.930
	37	*V. gigantis*	2.000
	42	*V. scophthalmi*	1.930
	43	*V. scophthalmi*	1.930

Shaded green, MALDI-TOF MS identifications that match assigned species identifications; shaded yellow, MALDI-TOF MS identifications that match assigned genus-level identifications only.
